# *Notes from the Field: *Undiagnosed Tuberculosis During Pregnancy Resulting in a Neonatal Death — United States, 2021

**DOI:** 10.15585/mmwr.mm7249a4

**Published:** 2023-12-08

**Authors:** Kathryn Miele, R. Bryan Rock, Sylvia M. LaCourse, David Ashkin, Lisa Y. Armitige, William Pomputius, Neela D. Goswami

**Affiliations:** ^1^Division of Birth Defects and Infant Disorders, National Center on Birth Defects and Developmental Disabilities, CDC; ^2^Division of Infectious Diseases, Hennepin Healthcare, Minneapolis, Minnesota; ^3^Departments of Medicine, Global Health, and Epidemiology, University of Washington, Seattle, Washington; ^4^Southeastern National Tuberculosis Center, Gainesville, Florida; ^5^Heartland National Tuberculosis Center, San Antonio, Texas; ^6^Division of Infectious Disease, Childrens Minnesota, Minneapolis, Minnesota; ^7^Division of Tuberculosis Elimination, National Center for HIV, Viral Hepatitis, STD, and TB Prevention, CDC.

In 2022, the World Health Organization reported 10.6 million new cases of tuberculosis (TB) globally. One third of these new cases were reported in women; however, pregnancy status was not included in these data.* CDC recently added pregnancy status to national TB reporting in the United States; however, because the number of U.S. TB cases during pregnancy is presumed to be low, adverse effects of TB on pregnancy and postpartum outcomes are likely not well characterized.^†^ A 2017 meta-analysis of 13 studies that included approximately 123,000 pregnancies from several countries found that TB disease during pregnancy was associated with increased odds of maternal morbidity and mortality, including hospital admission, anemia of pregnancy, cesarean birth, miscarriage, preterm birth, low birthweight, and neonatal TB (*1*). TB diagnosis during pregnancy might be delayed because of overlap in symptoms of TB with those of pregnancy, as well as clinician reluctance to use chest radiography during pregnancy.^§^ Perinatal TB is a life-threatening illness, with a congenital and neonatal TB mortality rate of approximately 50% (*2*), highlighting the importance of diagnosing and treating TB before and during pregnancy. This report describes a case of fatal neonatal TB after successful in vitro fertilization in 2021.

## Investigation and Outcomes

The infant’s mother underwent in vitro fertilization for infertility in her home country of India, which accounted for 27% of global TB incidence in 2022^¶^; she returned to the United States 1 month before delivery. During U.S. prenatal visits, she experienced insufficient weight gain, hyperemesis, and chronic cough, which was attributed to gastroesophageal reflux disease. Results for standard pregnancy laboratory tests were normal; no test for TB infection was performed. The mother experienced premature rupture of membranes at 33 weeks’ gestation followed by an uncomplicated spontaneous vaginal delivery of a healthy-appearing newborn and a normal-appearing placenta.

The newborn had 1- and 5-minute Apgar scores of 7 of 10 and 9 of 10, respectively, and weighed 5 lbs 6.7 oz (2,460 g) (90th percentile for gestational age). After receiving inpatient care for prematurity, the newborn was discharged home on the 14th day of life. However, shortly after hospital discharge, the infant developed labored breathing, became progressively ill, and was readmitted 4 days later (the 18th day of life) in septic shock, which was managed with endotracheal intubation and admission to an intensive care unit. Chest radiography demonstrated overall ground-glass–appearing infiltrates, suggesting inflammation, and loss of lung volume. On the basis of these findings, the mother’s chronic cough, and her origin from a country with high TB incidence, pulmonary TB was suspected. The infant’s gastric aspirate samples contained acid-fast bacilli on smear microscopy (an indicator of pulmonary TB) and grew *Mycobacterium tuberculosis* in culture. TB treatment** was commenced on the 22nd day of life. Initially, the infant’s condition improved, but 12 days after the diagnosis of TB, a pneumothorax was identified in the context of sudden respiratory deterioration. Respiratory treatments were not effective, and in alignment with the family’s wishes, support was withdrawn with institution of comfort measures. The infant died on the 42nd day of life of TB-related respiratory failure.

The mother’s chest radiograph demonstrated bilateral reticular nodular opacities. Acid-fast bacilli were identified on sputum smear microscopy, and a sputum sample tested positive for *M. tuberculosis* by polymerase chain reaction; a sputum culture was also positive. The mother recovered while completing a full course of treatment for drug-susceptible pulmonary TB, the same treatment that would have been recommended if a diagnosis had occurred during pregnancy. The only other household contact was determined not to have TB disease or latent TB infection after evaluation. This activity was reviewed by CDC, deemed research not involving human subjects, and was conducted consistent with applicable federal law and CDC policy.^††^

## Preliminary Conclusions and Actions

Although TB disease typically affects the lungs, it can involve any system, including the reproductive system, which can be affected in the absence of pulmonary findings (*3*). TB of the female reproductive system can cause infertility, pain, a pelvic mass, or menstrual disorders (*3*). Diagnosis requires a high index of suspicion for TB when a person from a country with endemic TB experiences genitourinary symptoms, including infertility. In India, TB is considered the likely cause of infertility in nearly one quarter (24.2%) of women with infertility (*3*). The sensitivity of chest radiography in detecting disease is 10%–75% in genitourinary TB (*4*). Ascertaining a diagnosis of TB during a female infertility evaluation should include consideration of pelvic organ imaging and specimen collection via laparoscopy and endometrial biopsy for acid-fast bacilli smear microscopy, polymerase chain reaction and culture for *M. tuberculosis*, and histology (*4*).

The fatal case reported here might have been avoided by TB prevention or TB treatment during the infertility evaluation or during pregnancy. This case underscores the importance of considering TB during an evaluation of women with infertility or a history of infertility if they are from a country with endemic TB. To reduce TB-associated morbidity and mortality, including congenital and neonatal TB, all persons, including those who are pregnant, should be considered for TB evaluation by assessing risk factors for TB infection (e.g., current or previous residence in a high TB-incidence country, a homeless shelter, or correctional facility) and risk factors for progression to TB disease if TB infection is present (e.g., diabetes, HIV infection, or substance use disorder)^§§^ (*5*) (Box).

BOX Selected groups with increased likelihood of infection with *Mycobacterium tuberculosis* and with increased risk for developing tuberculosis disease if infected*^,†,§^

**Groups with increased likelihood of infection with *Mycobacterium tuberculosis***

- Household contacts of or persons with recent exposure to an active tuberculosis case
- Immigrants from countries with a high tuberculosis incidence (>20 cases per 100,000 population)
- Residents and employees of high-risk congregate settings (e.g., homeless shelters or correctional facilities)
- Mycobacteriology laboratory personnel

**Groups with increased likelihood of developing tuberculosis disease if infected^§^**

- Children aged <5 years
- Persons with clinical predisposition (e.g., diabetes, HIV infection, receipt of immunosuppressive therapy, substance use disorder, or silicosis)
- Persons with abnormal chest radiograph consistent with previous tuberculosis disease

* https://doi.org/10.1093/cid/ciw778

^†^ https://doi.org/10.1542/9781610020886

^§^ Screening for persons at low risk is not recommended. The information in this box does not differentiate among likelihood or levels of risk for progression.

## Footnotes

*
https://www.who.int/news-room/fact-sheets/detail/tuberculosis

†
https://www.cdc.gov/tb/programs/rvct/instructionmanual.pdf

§
https://doi.org/10.1097/AOG.0000000000003890

¶
https://www.who.int/teams/global-tuberculosis-programme/tb-reports/global-tuberculosis-report-2023/tb-disease-burden/1-1-tb-incidence

** The infant’s treatment course was complicated by necrotizing enterocolitis, which precluded the administration of standard oral isoniazid (INH). Because intravenous INH could not be located in the United States, the infant received an intravenous regimen of meropenem, levofloxacin, linezolid, and rifampin.

†† 45 C.F.R. part 46.102(l)(2), 21 C.F.R. part 56; 42 U.S.C. Sect. 241(d); 5 U.S.C. Sect. 552a; 44 U.S.C. Sect. 3501 et seq.

§§
https://doi.org/10.1542/9781610020886
